# Pro-Inflammatory Effect of Gliadins and Glutenins Extracted from Different Wheat Cultivars on an In Vitro 3D Intestinal Epithelium Model

**DOI:** 10.3390/ijms22010172

**Published:** 2020-12-26

**Authors:** Francesca Truzzi, Camilla Tibaldi, Anne Whittaker, Silvia Dilloo, Enzo Spisni, Giovanni Dinelli

**Affiliations:** 1 Department of Agricultural and Food Sciences, Alma Mater Studiorum–University of Bologna, 40127 Bologna, Italy; camilla.tibaldi2@unibo.it (C.T.); whittaker.anne@gmail.com (A.W.); silvia.dilloo2@unibo.it (S.D.); giovanni.dinelli@unibo.it (G.D.); 2 Department of Biological, Geological, and Environmental Sciences, Alma Mater Studiorum–University of Bologna, 40126 Bologna, Italy; enzo.spisni@unibo.it

**Keywords:** *Triticum aestivum*, modern and old genotypes, total proteins, gluten strength, innate immunity, cytotoxicity, Caco-2 cells, U937 monocytes

## Abstract

There is a need to assess the relationship between improved rheological properties and the immunogenic potential of wheat proteins. The present study aimed to investigate the in vitro effects of total protein extracts from three modern and two landrace *Triticum aestivum* commercial flour mixes, with significant differences in gluten strength (GS), on cell lines. Cytotoxicity and innate immune responses induced by wheat proteins were investigated using Caco-2 monocultures, two dimensional (2D) Caco-2/U937 co-cultures, and three dimensional (3D) co-cultures simulating the intestinal mucosa with Caco-2 epithelial cells situated above an extra-cellular matrix containing U937 monocytes and L929 fibroblasts. Modern wheat proteins, with increased GS, significantly reduced Caco-2 cell proliferation and vitality in monoculture and 2D co-cultures than landrace proteins. Modern wheat proteins also augmented Caco-2 monolayer disruption and tight junction protein, occludin, redistribution in 3D co-cultures. Release of interleukin-8 into the cell medium and increased U937 monocyte migration in both 2D and 3D co-cultures were similarly apparent. Immuno-activation of migrating U937 cells was evidenced from cluster of differentiation 14 (CD14) staining and CD11b-related differentiation into macrophages. The modern wheat proteins, with gluten polymorphism relatedness and increased GS, were shown to be more cytotoxic and immunogenic than the landrace wheat proteins.

## 1. Introduction

Wheat is one of the major staple crops worldwide, with approximately 95% of total production being represented by *Triticum aestivum* L. ssp. *aestivum* (allohexaploid: AABBDD, 2*n* = 42), generally referred to as common (bread, soft) wheat. The seed storage proteins (8–15% of the total flour weight) are important determinants of end-product quality, with improved bread-making properties largely attributable to the D genome [1,2]. Seed proteins can be classified on the basis of solubility into albumins and globulins (20–25% of the total protein content) as well as gluten proteins (75–80% of the total protein content), comprised of monomeric gliadins (ω5-, ω1,2-, α-, and γ-gliadins) and polymeric glutenins (high- and low-molecular-weight glutenin subunits (HMW-GluS, LMW-GluS)), respectively. However, in recent decades, the ingestion of wheat proteins has been increasingly associated with clinical disorders, such as celiac disease (CD), wheat allergy (WA), and non-celiac gluten sensitivity (NCGS), also known as non-celiac wheat sensitivity (NCWS), which are becoming epidemiologically more relevant on a global scale [2,3,4,5].

Gliadins, in particular the α- and ω-gliadins, have been shown to the most be potent reactive inducers of CD, an autoimmune inflammatory disorder [1]. Collectively, potent reactive components in WA (Baker’s asthma, atopic dermatitis, urticaria, and anaphylaxis) include α- and ω-gliadins, glutenins, as well as amylase-trypsin inhibitors (ATIs), serpins, and wheat lipid transfer proteins associated with the albumin/globulin fraction [1]. Though the pathophysiology of NCWS is still poorly understood, gluten (particularly gliadins) and ATIs are among the proteins implicated in gastrointestinal inflammation [2,4,6,7]. Interestingly, several highly immunogenic α-gliadins are encoded by the D genome [8], thereby rendering *T. aestivum* more immuno-reactive compared to *Triticum turgidum* L. spp. *durum* (tetraploid AABB, 2*n* = 28) [9]. Given the increasing prevalence of both CD and NCWS, coinciding with the release of new wheat hybrids, for some experts these disorders are definitely the consequence of modern wheat cultivars [3]. The premise was that breeding goals for higher yield and improved technological parameters during the “Green Revolution” (period around 1950) may have inadvertently contributed to a higher immune-stimulatory potential of modern wheat cultivars compared to old wheat cultivars (landraces and heritage genotypes cultivated prior to the “Green Revolution”). Faced with a rapidly evolving public health issue in the form of wheat-related disorders combined with the lack of scientific evidence regarding the higher immune-stimulatory potential of modern wheat cultivars [3], research focus has been directed toward the study of gluten proteins in old and modern genotypes of both *T. aestivum* and *T. durum*.

Agriculturally based research on gluten proteins in old and modern genotypes has encompassed the study of changes principally on the levels of genotype, environment (including agronomic practices), and technological parameters, respectively [1,10,11]. More recent studies have investigated the in vitro immunogenicity of glutens between landraces and modern genotypes [2,9,12,13,14,15], with new multidisciplinary approaches favoring comparative studies between in vitro immunogenicity and technological properties [16]. Overall, to date, results from the aforementioned studies have shown immense variation in the immunogenic potential in both old (landraces) and modern genotypes. Studies of “adverse effects” have often been reported using isolated protein fractions for comparison and, although results are less confounded by potential environmental effects, [17], the latter cannot be excluded when attributing genotype-based differences. Both environmental parameters and agronomic practices have been shown to exert significant effects on protein composition and of particular relevance is the impact of higher nitrogen fertilizer treatments, used in modern wheat cultivation, in augmenting immune-stimulatory ω- and α-gliadins [1,3,11]. Further studies are urgently required on a wider range of genotypes of ancient (old) and modern wheat species [17].

There is also a requirement for multidisciplinary studies to include technological features such as gluten strength (GS), an important parameter of modern wheat breeding determining end-use product destination in baking industries [16]. GS is the ability of the proteins to form a satisfactory network in terms of continuity and strength (thereby determining end-use quality). Of note, wheat products are rarely labeled and are sold as combinations (mixtures) of many wheat varieties [1]. The study of the immunogenic potential in commercially available wheat mixtures is of interest to the public, in particular NCWS sufferers who seek to lower the amount of reactive wheat components and subjects who may be predisposed to either NCWS or CD.

The immunogenic potential of proteins on the intestinal epithelium can be successfully assessed using various in vitro cell cultures, based on the resemblance of physiological traits of the respective cell lines with their target/original tissue [18]. To date, the Caco-2 cell monolayer, a human colon intestinal carcinoma cell layer, is considered the “gold standard” of in vitro models in mimicking or simulating the intestinal epithelium for testing the remedial potential of functional food extracts, as well as cytotoxic effects attributable to reactive gluten proteins [18,19]. Unlike CD, in which gluten induces both adaptive (specific) and innate (nonspecific) immune responses, the innate immune response is exclusive to NCWS [4,5,6]. Hence, investigating pro-inflammatory cytokines and proteins, characterizing the innate immune response, would be relevant toward the selection of “less cytotoxic” candidate genotypes for both NCWS individuals and those genetically predisposed to wheat sensitivity and CD. To the best of our knowledge, only a single published paper has assessed the effect of gliadin proteins of 12 old and modern *T. aestivum* genotypes using the cell line HCT116 [15].

Given the requisite for assessing innate immune responses to wheat proteins on the basis of improved technological parameters (a result of modern wheat breeding), the present study was aimed at investigating total protein extracts from three modern and two old, commercially available flour mixes (selected on the basis of significant differences in GS) on the Caco-2 cell line. Given that the intestinal barrier is comprised of structural epithelial cells together with immune cells (monocytes, fibroblasts), with respective “cross-talk” between the latter, the study was extended to include innate immune responses induced by wheat proteins in 2D co-cultures of Caco-2/U937 (monocytes) as well as 3D co-cultures of Caco-2/U937/L929 (fibroblasts).

## 2. Results

### 2.1. Protein Content, Composition, and Rheological Properties

The five wheat flour samples were firstly analyzed for crude protein content and rheological properties. Ash and crude protein content were significantly higher in the heritage flour mixes, Lacollina_A and Virgo_A, than in the modern flour mixes, Biscottiero_M, Panificabile_M, and Diforza_M (Table 1). The moisture percentage was equivalent between the samples and wet gluten content, similar to crude protein content, was significantly higher in the two landrace flour mixes than in the three modern wheat flours (Table 1).

The flour samples of the three modern flour mixes were specifically selected based on significant differences in baking (gluten) strength. The end-use quality classification is represented by confectionary wheat (“frumento biscottiero”), ordinary bread-making wheat (“frumento panificabile”), and improved wheat (“frumento di forza”), respectively. Hence, the wheat flours, based on the different baking (gluten) strengths (Table 1), are referred to as Biscottiero_M, Panificabile_M, and Diforza_M, respectively. The alveograph dough baking value (W) is a widely used parameter to determine technological end-use suitability, with a lower W denoting a lower quality baking complex or gluten strength. The W value in the heritage flour mixes, Lacollina_A and Virgo_A, was significantly lower than those of the modern variety flour mixes (Table 1). Similarly, the alveograph P/L index (dough tenacity or strength / dough extensibility index) is also a commonly used parameter to determine end-product use and is calculated from the ratio between P (GS) and L (extensibility), with higher P/L values being representative of strong gluten flours with lower extensibility (stretch). Only between the three modern varieties flour mixes was P/L positively correlated to increasing W (Table 1). P/L values of Lacollina_A and Virgo_A were comparable to that of Panificabile_M.

The proteins were extracted and the albumin and globulin fraction (A + G), as well as the gluten fraction (gliadins and glutenins), were analyzed separately by SDS-PAGE (Figure S1). The protein profiles of the different flour samples differed both in the number of bands and in band intensity. In Panificabile_M, Biscottiero_M, and Diforza_M 17 bands were detected in the A + G fraction whereas 16 bands were detected in Virgo_A and 15 in Lacollina_A, respectively (Figure S1A). For the gluten fraction, 22 bands were detected in the two landrace flour mixes and Biscottiero_M (Figure S1B). Instead, 24 and 20 bands, respectively, were detected in Panificabile_M and Diforza_M. Moreover, certain bands were distinguishable for the different flour mixes. The bands at 100, 94, 89, 84, and 82 kDa were detected exclusively in the landraces, whereas a band at 110 kDa was evident only in Panificabile_M and Biscottiero_M and a band at 99 kDa only in Diforza_M, respectively (Figure S1B).

As the objective of the study was to investigate the in vitro effect of total proteins on intestinal cell models, it was important to establish whether potential differences in the effects on the cells between flour mixes were attributable to possible differences in their respective protein composition. For this reason, dendrograms of hierarchical relatedness based on A + G and gluten polymorphisms were constructed. The dendrograms would indicate distinctive groupings or clusterings between the more similar and divergent flour mixes, respectively. Therefore, the information pertaining to the total number of bands and molecular weight size distribution for each sample was used to construct dendrograms of hierarchical relatedness.

**Figure 1:**
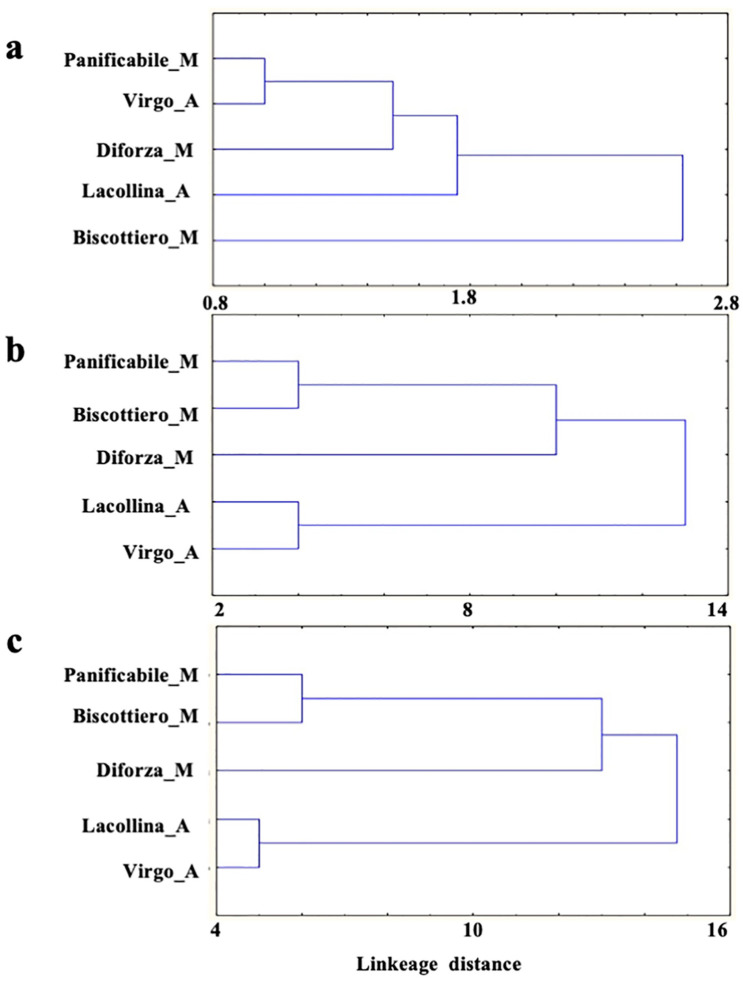
Dendrogram of hierarchical relatedness based on (**a**) albumins and globulins, (**b**) gliadins and glutenins, and (**c**) total protein polymorphisms between in three commercially available modern wheat mixes (denoted by M) and two old wheat mixes (denoted by A).

For A + G, two clusters were evident, with Biscottiero_M being the most divergent, forming a single cluster (Figure 1a). The remaining samples were divided into three subclusters, with Panificabile_M and Virgo_A being more closely related. Based on the glutens, Figure 1b indicates two clusters, distinctive for the heritage and modern flour mixes, respectively. Within the two clusters, the closest degree of relatedness was between Lacollina_A and Virgo_A and between Biscottiero_M and Panificabile_M, respectively. Diforza_M formed a separate subcluster within the modern flour cluster. Combining all the protein fractions, the two clusters of relatedness were present for the heritage and modern flours, with a higher degree of relatedness evident for Lacollina_A and Virgo_A than that for Biscottiero_M and Panificabile_M, within the two clusters, respectively (Figure 1c). Within the cluster of modern flour mixes, Diforza_M was more divergent.

### 2.2. In Vitro Effects of Modern and Old Common Wheat Protein Mixes on Caco-2 and U937 Cell Lines

Given that GS (Table 1) and gluten relatedness (Figure 1b,c) were distinctive for old and modern flour mixes, the objective was then to investigate whether GS was associated with differences in the immuno-stimulating potential of the protein extracts by investigating the in vitro effect on intestinal cell models.

A 40-µg/mL dose of protein extract that included all protein fractions, representative of the in vivo composition, was selected for each of the five samples, respectively, and used as a standard content for all cell culture experiments. Preliminary experiments with 20-μg/mL wheat protein extracts showed no effect on cell proliferation, whereas 80 μg/mL total protein resulted in extensive damage to the cells, resulting in more than 80% inhibition on proliferation. The 40-µg/mL dose, incubated with the Caco-2 cells for 24 h, affected cell proliferation moderately and was, therefore, considered a concentration guaranteed to distinguish sample-based differences in the cell culture experiments. The decision to use all representative protein fractions, rather than single fractions, was to include all potential immunogenic epitope proteins. All experimental analyses on the response of the cell lines to the total protein in the five samples were conducted in triplicate.

**Figure 2:**
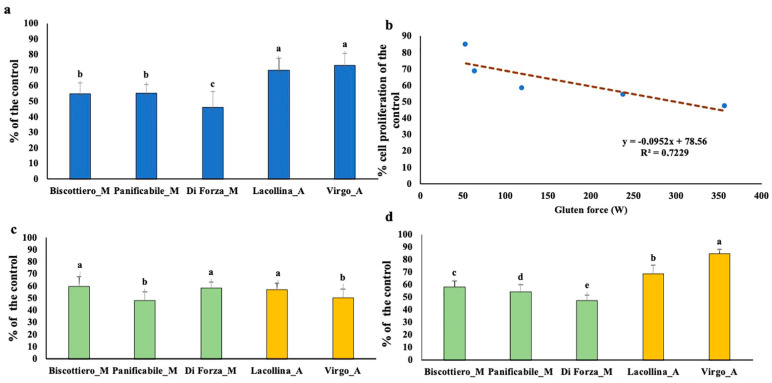
Cell proliferation (as a percentage of the untreated control) of Caco-2 cells after a 24 h exposure to (**a**) 40 µg/mL total protein (albumins + globulins + gliadins + glutenins), (**c**) 40 µg/mL albumins + globulin, and (**d**) 40 µg/mL gliadins and glutenins from three commercially available modern wheat mixes (denoted by M) and two old wheat mixes (denoted by A). The designation of the letters a, b, c, and d represents significant differences between varieties as determined by one-way ANOVA at the 95% confidence level (*p* ≤ 0.05). (**b**) is the correlation between gluten strength and effect on cell proliferation of the total protein extract.

In the preliminary Caco-2 monoculture experiments, cell proliferation after exposure to the five wheat protein samples was examined and expressed as a percentage of the untreated control. Total protein of both heritage mixes (Lacollina_A and Virgo_A) reduced cell proliferation of the Caco-2 cells by ca 20–30%, whereas a greater inhibitory effect was increasingly evident for Biscottiero_M/Panificabile_M (40–50%) and Diforza_M (50–60% reduction), respectively (Figure 2a). Interestingly, there was a significant inverse correlation between decreasing inhibitory effect of total protein on Caco-2 cell proliferation and increasing GS of the five samples (Figure 2b). Given that no studies have investigated the reactivity of ATI albumins between heritage and modern wheat genotypes [1], the water soluble (A + G) and insoluble gluten fractions were individually examined on Caco-2 cell proliferation for the five samples.

Noteworthy, there was a significant inhibitory effect, resulting in a ca 40–50% reduction in cell proliferation after exposure to A + G from both the old and modern flour mixes (Figure 2c). Of great interest was that the two more closely related samples for A + G, namely Panificabile_M and Virgo_A (Figure 1a), were the two samples with the significantly greater inhibitory effect on Caco-2 cell proliferation (Figure 2c). The gluten fraction showed more distinctive GS-based differences on cell proliferation (Figure 2d). A negligible effect was evident for Virgo_A (ca 20% reduction), with an increasing effect, in order of flour mix, shown for Lacollina_A, Biscottiero_M-Panificabile_M, and, finally, Diforza_M (40–50%), more closely reflecting the situation when total protein was administered.

**Figure 3:**
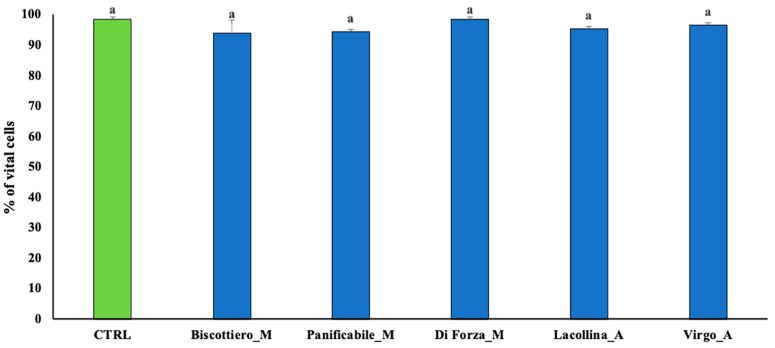
Cell vitality of U937 cells after a 24 h exposure to 40 µg/mL total protein (albumins + globulins + gliadins + glutenins) from three commercially available modern wheat mixes (denoted by M) and two old wheat mixes (denoted by A). The designation of the letters a, b, c, and d represents significant differences between varieties as determined by one-way ANOVA at the 95% confidence level (*p* ≤ 0.05).

Exposure of the monocyte cell line, U937, to 40 µg/mL total protein for 24 h was also investigated (Figure 3). No effect on the cell vitality was observed between the untreated control and five samples.

It was important to firstly establish whether and to what extent the wheat proteins affected the vitality of the U937 cells in monoculture before using these monocytes in the 2D and 3D co-cultures. In the latter models, the objective was to investigate the migration capacity of the U937 cells in response to “cross-talk signals” induced by the wheat proteins on the Caco-2 cells. Hence, given that vitality was not affected, based on the results of Figure 3, migration potential could then be investigated in the 2D co-cultures.

### 2.3. Wheat Protein Effects on Vitality, Migration, Interleukin-8, Prostaglandin E2, and Occludin Expression in Two-Dimensional Caco-2/U937 Co-Cultures

To simulate pro-inflammatory signaling “cross talk” from epithelial cells to associated migratory monocytes, Caco-2 and U937 cells were analyzed in 2D co-culture. The effect of total protein from the five samples on both Caco-2 cell vitality and U937 migration was first investigated. Since lipopolysaccharide (LPS) is a well-documented inducer of inflammation, LPS-treated Caco-2 cells were included as an additional comparative control. Thereafter, the “upstream” presence of pro-inflammatory markers in the medium secreted by Caco-2, in response to reactive wheat proteins, to induce migration of U937 was investigated.

**Figure 4:**
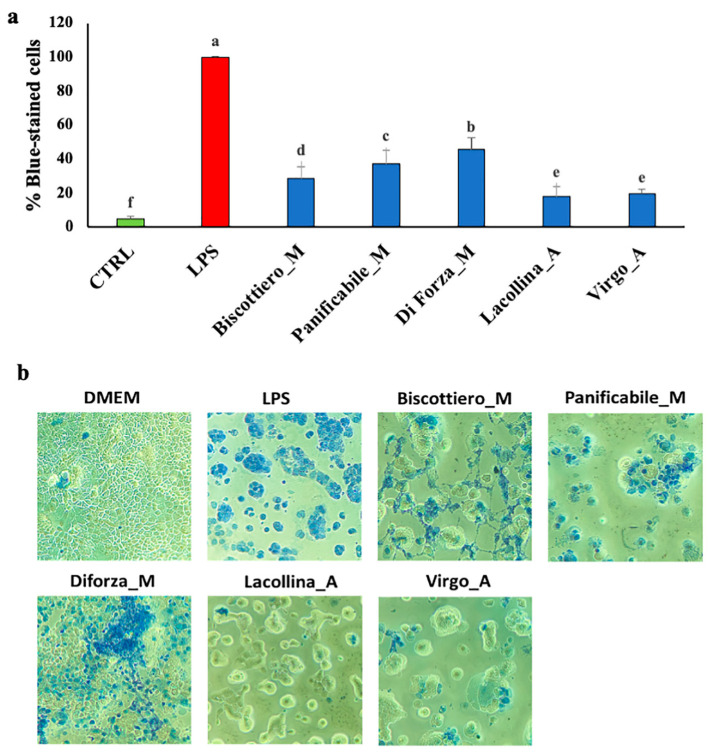
Cell vitality of Caco-2 cells in co-culture (**a**) showing the percentage of trypan blue-stained cells and (**b)** phase contrast microscopy (40× magnification) images of the Caco-2 cells after a 24 h exposure either 1 µg/mL LPS or 40 µg/mL total protein (albumins + globulins + glaidins + glutenins) from three commercially available modern wheat mixes (denoted by M) and two heritage wheat mixes (denoted by A). The designation of the letters a, b, c, and d represents significant differences between varieties as determined by one-way ANOVA at the 95% confidence level (*p* ≤ 0.05).

Overall cell vitality of Caco-2 in response to total protein exposure for 24 h was expressed in relation to maximum damage (set at 100%) induced by LPS-treated cells (Figure 4a), with a highest visual representation of blue (dead) cells attributable to inflammatory events causing cell permeability (Figure 4b). The presence of vital cells was over 90% in the untreated control with a minimal presence of blue-stained cells (Figure 4a,b). Both heritage mix proteins (Lacollina_A and Virgo_A) induced negligible cell permeability (ca 20%) compared to the modern genotype proteins (ca 30–45) (Figure 4a,b). In order of an increasing cell permeability-induced effects were the protein extracts of Biscottiero_M, Panificabile_M, and Diforza_M, respectively. Differences in cell vitality in the 2D cultures (Figure 4a) and cell proliferation in the monocultures (Figure 2a) attributable to the five wheat samples were comparable and associated with GS (Figure 2b).

**Figure 5:**
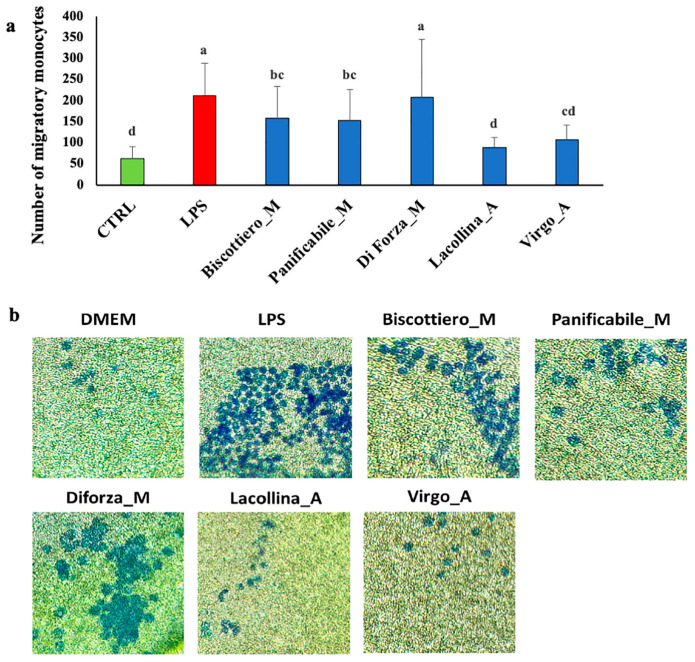
(**a**) Number of migratory U937 monocytes and (**b**) phase contrast microscopy (40× magnification) images of cells stained with toluidine blue thiazine dye after a 24 h exposure of Caco-2 cells in co-culture to either 1 µg/mL LPS or 40 µg/mL total protein (albumins + globulins + gliadins + glutenins) from three commercially available modern wheat mixes (denoted by M) and two heritage wheat mixes (denoted by A). The designation of the letters a, b, c, and d represents significant differences between varieties as determined by one-way ANOVA at the 95% confidence level (*p* ≤ 0.05).

Cell migration of U937, in response to “signal cross talk” emitted from Caco-2, was analyzed and the maximum migration of U937 cells was observed in response to LPS-treated Caco-2 cells (Figure 5a), as visualized by toluidine blue thiazine dye (Figure 5b). Minimal cell migration of U937 was induced by untreated Caco-2 control cells. Similar to the control, U937 migration was minimal in response to Caco-2 cells treated with both heritage mixes (Lacollina_A and Virgo_A), respectively (Figure 5a,b). Lacollina_A and Virgo_A induced minimal U937 migration (Figure 5a,b). The total protein from all three modern flour variety mixes exerted a higher Caco-2-induced migratory effect on U937 than the two old variety mixes, with U937 migration of Diforza_M-treated Caco-2 cells comparable to that exerted by the pro-inflammatory stimulant, LPS (Figure 5a,b).

**Figure 6:**
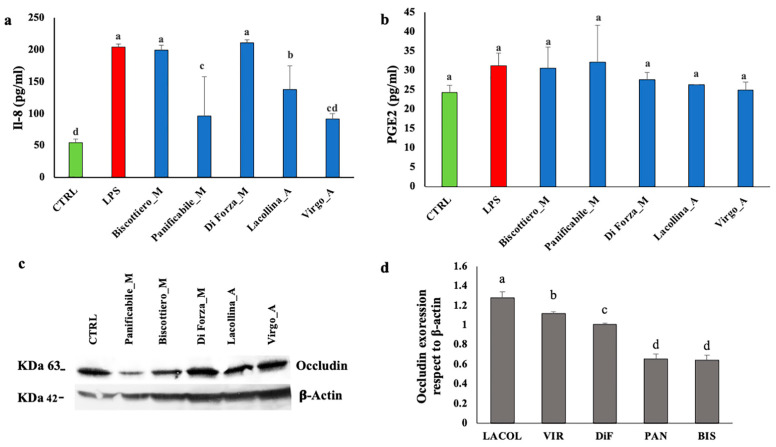
(**a**) Interleukin-8 (IL-8) and (**b**) prostaglandin E2 PGE-2 content in the cell medium after a 24-h exposure of Caco-2 cells in co-culture to either 1 µg/mL LPS or 40 µg/mL total protein from three commercially available modern wheat mixes (denoted by M) and two heritage wheat mixes (denoted by A). (**c**) Western blot of occludin and (**d**) occludin expression with respect to β-actin in Caco-2 cells. The designation of the letters a, b, c ,and d represents significant differences between varieties as determined by one-way ANOVA at the 95% confidence level (*p* ≤ 0.05). Ctrl = Control; LACOL = Lacollina_A; VIR = Virgo_A; DiF = Diforza_M; PAN = Panificabile_M; BIS = Biscottiero_M.

Interleukin-8 (IL-8) is a stress-responsive pro-inflammatory chemokine, facilitating an early innate immune response [20]. Given that IL-8 release from epithelial cells is strongly chemotactic for all known types of migratory immune cells [20] and has been documented to increase in gliadin-treated Caco-2/TC7 cells [21], IL-8 in the cell medium was measured. LPS-treated Caco-2 cells induced the increase in IL-8, which was released into the medium (Figure 6a). Protein extracts of Diforza_M and Biscottiero_M induced equivalent IL-8 levels to that of LPS. Protein extracts of Panificabile_M induced significantly lower levels of IL-8 compared to the former modern flour proteins, although there was a high degree of variation (Figure 6a). Virgo_A-treated Caco-2 cell lines showed IL-8 levels that were not significantly different from the control, indicating that wheat protein from this landrace flour mix did not induce IL-8 production. In contrast to the Virgo_A-treated Caco-2 cell lines, cells treated with landrace Lacollina_A produced more IL-8 but with a large degree of variation. Reasons for the variability in this sample are not known.

Prostaglandin E2 (PGE2), synthesized as a result of pro-inflammatory-induced cyclooxygenase-2 (COX-2) expression, is a lipid mediator, able to regulate the migration of several immune cells, particularly those involved in innate immunity such as macrophages [22]. Given that both COX-2 and PGE2 expression were previously reported to have been upregulated in gliadin-treated Caco-2/TC7 cells [21], PGE2 was measured in the cell medium. There was no induction of PGE2 above the untreated baseline control levels in either the LPS-treated or protein extract-treated Caco-2 cells (Figure 6b). This result indicated that the pro-inflammatory-inducing conditions by both LPS and the wheat proteins did not result in the expression in PGE2 by the Caco-2 cells within the 24-h time frame of the present study.

The maintenance of epithelial barrier integrity is regulated by tight junction (TJ) proteins, of which occludin is suggested to be important in the assembly and maintenance of TJs [23]. Given that gliadins have previously been shown to downregulate occludin protein expression in Caco-2 lines and enhance cellular permeability [24,25,26], occludin protein expression in the Caco-2 lines induced by the five samples was investigated. Figure 6c shows a Western blot of occludin and β-actin, which was used as an internal control. Occludin was then normalized to β-actin and the control set at a value of 1.0 (Figure 6d). In Caco-2 cells exposed to the two landrace flour mixes, occludin protein content was significantly higher than that expressed in the cells that were exposed to the modern wheat flour mixes. Only Biscottiero_M and Panificabile_M proteins induced the reduction of occludin in Caco-2 cells.

### 2.4. Wheat Protein Effects on Epithelial Structure, Tight Junction Protein Localization, Monocyte Migration, and Macrophage Differentiation in Three-Dimensional Caco-2/U937/L929 Co-Cultures

The 3D co-culture systems provide more physiologically and structurally relevant in vitro models for studying inflammation, being more reflective of in vivo cellular responses [27]. In the present study, U937 monocytes and L929 fibroblasts were embedded in a collagen layer on a Transwell filter insert and Caco-2 cells were seeded on top, to simulate the intestinal mucosa with the epithelial monolayer situated above an extracellular matrix (ECM)-rich lamina propria, consisting of stromal cells, collagens, monocytes, and various immune cells.

**Figure 7:**
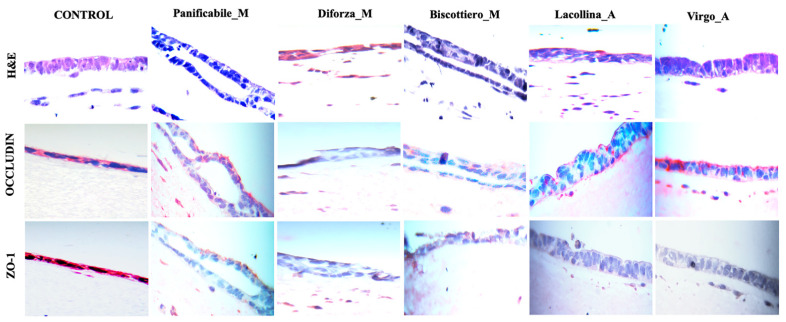
Hematoxylin and eosin (H & E), occludin, and zonula occludin-1 ZO-1 staining of 3D intestinal equivalents (Caco-2/U937/L929 co-cultures) exposed to no added protein (control) or 40 µg/mL total protein from three commercially available modern wheat mixes (denoted by M) and two heritage wheat mixes (denoted by A) for a period of 24 h. For occludin and ZO-1 staining, fast red was used as a chromogen and nuclei were counterstained with eosin. The magnification was ×40.

Hematoxylin and eosin (H & E) staining of the 3D intestinal equivalents (Figure 7) showed preservation of the Caco-2 columnar cells forming a tight and regular monolayer in both the control and models exposed to the heritage flour mixes (more specifically, Virgo_A). Instead, in the reconstituted models exposed to the modern flour mixes, Biscottiero_M and Panificabile_M, monolayer disruption and detachment from underlying ECM was evident (Figure 7). Diforza_M exposure also caused disruption of the monolayer, with more nonspecific eosin staining compared to the remaining samples.

Results are in agreement with reduced cell proliferation and vitality results of Caco-2 in monoculture and 2D co-culture, respectively, in relation to gluten strength (Figure 2a,b; Figure 4a,b).

The interaction between phosphorylated occludin and the intracellular scaffolding protein, zonula occludin-1 (ZO-1), is essential for TJ integrity of epithelial cells. Given that gliadin-induced effects have been shown to result in the downregulation and/or redistribution of occludin and ZO-1 with a consequent increased monolayer permeability [24,25,26], occludin and ZO-1 distribution were investigated. Occludin localization in the Caco-2 cell monolayer was uniform in the control (Figure 7). Uniform occludin staining, specifically along the apical region of the Caco-2 cells exposed to proteins from Virgo_A, was observed. Occludin staining was similarly present in Caco-2 cells exposed to Lacollina_A, but appeared less uniform. Although a slight downregulation of occludin protein was evident after exposure to the modern flour mixes (compared to the heritage mixes, Figure 6c,d), occludin staining was localized in the Caco-2 cell layer of Biscottiero_M and Panificabile_M but was more diffuse within the cells (Figure 7). In cells treated with Diforza_M no red staining was discernable (Figure 7). Similar to occludin, intense and uniform ZO-1 staining was distinct in the control (Figure 7). Discontinuous ZO-1 staining was evident in the images of models exposed to Panificabile_M. However, no staining of ZO-1 was evident in the remaining models exposed to wheat proteins (Figure 7). The lack of ZO-1 staining (detection) suggested that this compound had been broken down in the Caco-2 cells and lost prior to the 24-h time point, coinciding with the termination of the experiment.

**Figure 8:**
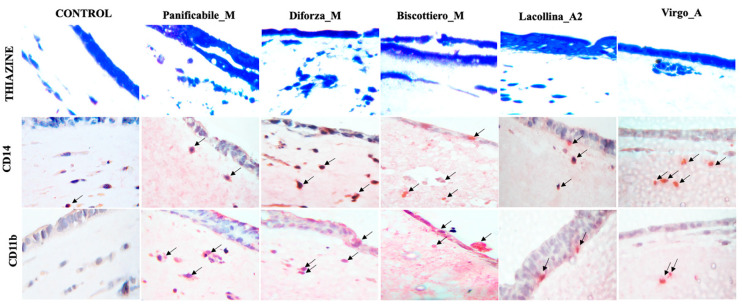
Thiazine staining as well as CD14 staining of U937 monocytes and CD11B U937 differentiated macrophages in 3D intestinal equivalents (Caco-2/U937/L929 co-cultures) exposed to no added protein (control) or 40 µg/mL total protein from three commercially available modern wheat mixes (denoted by M) and two heritage wheat mixes (denoted by A) for a period of 24 h. For CD14 and CD11b staining, fast red was used as a chromogen and nuclei were counterstained with eosin. Arrows indicate the presence of monocytes (CD14 positive cells) and macrophages (CD11b positive cells). The magnification was ×40.

Thiazine, a nuclear stain, was used to localize cellular components within the reconstituted intestinal mucosa. In the control, the staining of the Caco-2 cell monolayer was evident as well as the staining of U937 and L929 cells within the ECM some distance below the Caco-2 monolayer (Figure 8). Pro-inflammatory stress causes migration of monocytes but also fibroblasts, which play a role in wound healing. In the reconstituted 3D models, exposure to wheat protein in all flour mixes caused the migration of cells within the ECM toward the Caco-2 cell layer, although the respective U937 and L929 cells were not distinguishable between them.

A greater number and degree of cell clustering was evident in models exposed to the modern genotype flour mixes (Figure 8), corroborating the increased migration evident from the 2D experimental co-cultures (Figure 5a,b). Verification of U937 monocyte activation and resultant migration, as well as differentiation into macrophages, was then evidenced by specific staining. CD14, a glycolipid-anchored membrane glycoprotein expressed on monocytes and macrophages, enables the identification of these cells. Pro-inflammatory inducers such as LPS have been shown to increase the CD14 expression on the surface of U937 monocytes [28]. Fast red-coupled CD14 staining was anticipated to predominantly stain activated U937 monocytes but did not preclude the staining of macrophage-differentiated U937 cells. Staining of activated monocytes was not evident in the control model, whereas in the models exposed to all wheat protein flours staining of U937 in the ECM was distinct (Figure 8). Evidence of migration was evident from presence of activated U937 cells close to the Caco-2 monolayer and, in the case of disrupted monolayer, U937 cells were observed directly within the disrupted Caco-2 cells, particularly discernable in the images of intestinal equivalents exposed to Diforza_M and Biscottiero_M (Figure 8).

U937 cells have been shown to be induced to differentiate into macrophages, identifiable by the expression of the monocytic differentiation marker CD11b and concomitant morphological changes [29,30]. For this reason, U937 cell differentiation into macrophages was also investigated. No CD11b staining was evident in the control. In the models exposed to the heritage flour mixes, a few macrophages were manifested, either at a distance from or just below the Caco-2 monolayer in Virgo_A and Lacollina_A, respectively (Figure 8). Macrophage staining was more abundant in the reconstituted models exposed to the modern flour mixes and the presence of the macrophages was observed both within the disrupted Caco-2 monolayer as well as within the simulated ECM-rich lamina propria below (Figure 8). A well-defined cell with macrophage morphology was evidenced as passing through the monolayer in the model treated with Biscottiero_M (Figure 8).

## 3. Discussion

Combining the requirement to examine the immunogenic potential of wheat genotypes on the basis of improved technological parameters together with that of expanding comparative studies between heritage and modern genotypes, the present study adopted a function-based approach by using cell lines (simulating the intestinal mucosa) to measure the effects *T. aestivum* protein extracts from commercially available wheat sources of modern and heritage flours in relation to GS. In the present study, flour mixes with increased GS (associated with the modern varieties) were more cytotoxic, resulting in a greater pro-inflammatory-induced reduction in cell proliferation of Caco-2 cells in monoculture, as well as in the vitality of Caco-2 cells and migration of U937 cells in 2D co-culture. These results were supported by 3D co-cultures, showing that the higher GS proteins induced a greater damage to Caco-2 monolayer integrity, increased TJ protein delocalization, as well as an increased activation U937 monocytes, and resultant differentiation into macrophages, compared to the heritage flour mixes. Of interest, modern genotypes are lower in gliadin content [11]. Given that the gliadins are documented as more immunogenic than the glutenins, it is paradoxical that the increased prevalence of both CD and NCWS appear to be associated with the consumption of modern genotypes [3]. For this reason, it is necessary to unravel the relationships between functional immunogenicity and technical/rheological properties.

Previous work demonstrated that increased GS in modern varieties was specifically associated with increased HMW-GluS contents [31,32,33,34] This would infer that the greater pro-inflammatory effects of higher GS wheat proteins were in some manner, either directly or indirectly, associated with the increasing prevalence of HMW-GS. The causative immunogenic proteins were not identified in the present study but the need to further investigate associations between the higher GS and associated immunogenicity was evidenced. In a very recent, comprehensive study by Pilolli et al. [16], 38 genotypes with a lower total gluten content (including both heritage and modern representatives) were selected from a total of 240 tetraploid *T. durum* genotypes. From that subset, five genotypes were then selected, based on a lower presence of gliadin epitopes as well as a lower GS (measured by the gluten index). The five genotypes were then subjected to in vitro protein digestion and potential toxicity analysis by in silico risk assessment, providing evidence for the existence of low toxicity candidate genotypes with medium to strong GS necessary for pasta making [16]. No analogous article exists for allohexaploid *T. aestivum* genoytpes, in which the situation is more complicated. Both the improved bread-making properties as well the presence of cytotoxic gliadins were shown to be present on the D genome of *T. aestivum* [1,2,8,9]. Given the complex genetic linkages between different classes of glutens and the presence of ω-gliadins on more than one chromosome [8], the positive technological properties co-exist with the potentially harmful gliadins. As a result, the cytotoxic effects on the cells in the present study, attributable to the modern flour mixes, may be due to the expression of toxic gliadin epitopes that are expressed with the glutenins on the D genome. In a single study using both older (released between 1920–1960) and modern (released 2012) *T. aestivum* genotypes to investigate in vitro effects on intestinal HCT116 cell lines it was shown that gliadin proteins isolated from the old varieties (released between 1920–1940) produced either slight or no change in oxidative markers and pro-inflammatory cytokines compared to the modern varieties [15]. The results of Gupta et al. [15] corroborated previous results [12] showing that, although both *T. aestivum* heritage and modern varieties contained gliadins that induced inflammation, the heritage varieties were less immunogenic.

The increased cytotoxic effects of the three modern (higher GS) flour mixes may not be solely attributable to the presence of the gliadins, but also directly related to the glutenin fraction (specifically HMW-GluS). There is far less information on the immunogenicity of glutenin proteins, invariably attributable to a far greater historic research investment in gliadin protein research. Previous work has shown that HMW-GS are immuno-stimulating in CD and are able induce an innate immune response in cell lines [35,36]. More recently, monoclonal antibodies have been developed to HMW-GS that are known to be immuno-stimulating in CD, with the objective toward quantifying CD toxic gluten in foods [37]. Given that the parameter of GS was shown to vary extensively both within landraces and modern varieties [16,38], we suggest that the study on *T. aestivum* be extended to examine the in vitro immunogenicity of a larger set of commercial wheat mixes specifically selected on the basis of GS. Such an approach would contribute toward the identification of lower immune-reactive *T. aestivum* genotypes, more suitable for individuals at risk of CD and NCWS.

From the present study, the albumin/globulin fraction reduced cell proliferation of Caco-2 cells in all samples analyzed, indicating a potential adverse effect of this fraction on intestinal cells. Although it was proposed that immunogenic ATIs (albumin proteins) may have been increased in modern wheat-breeding programs [7], to the best of our knowledge no studies yet exist comparing heritage and modern varieties. In this preliminary study there was no indication that the albumin/globulin fraction of the modern flour mixes reduced Caco-2 cell proliferation to a greater extent than that of the landrace flour mixes. Interestingly, the present results showed that two samples (one modern and one old wheat flour mix, respectively), with the closest degree of relatedness from the A + G fraction dendrogram, reduced Caco-2 cell proliferation more significantly than the remaining three flour mixes. However, when administered with the gluten fraction, in physiologically relevant in vivo proportions, there was no evidence to suggest that these two samples, which reduced cell proliferation to a significantly greater extent, were distinctive from the remaining varieties in influencing the vitality of Caco-2 cells and migration of U937 cells in co-culture.

To verify a specific pro-inflammatory causative effect on Caco-2 cellular proliferation and vitality necessitated investigating the presence of standard specific upstream inflammatory markers. The present results showed a pro-inflammatory-induced expression of IL-8, with comparable levels between LPS-treated and protein (modern variety) extract-treated Caco-2 cells. IL-8 expression, the most abundant inflammatory cytokine produced by the intestinal epithelium and extensively used as a screening tool to detect induction of an inflamed epithelial state [39], corroborated previous work on Caco-2 cells in response to immunogenic gliadin proteins [21,40]. The subsequent secretion of IL-8, a potent chemo-attractant of immune cells [20], into the Caco-2 cell medium was a likely contributory factor in stimulating U937 migration. Higher levels of migration were associated with increased IL-8 in two of the high gluten strength (modern variety) flour mixes, whereas no induction of expression was reported for one low gluten strength (landrace). The comparative potential was marred by the large variation in the remaining two samples. A more definite conclusion would necessitate a greater sample size.

Contrary to expectation, there was no increase in PGE2 levels in the LPS-treated and protein extract-treated Caco-2 cells. Similar to IL-8, COX-2 expression, responsible for the synthesis of PGE2 protein, is triggered by mitogen-activated protein kinase (MAPK) and nuclear factor kappaB, (NF-kB) transcription pathways in response to pro-inflammatory stimuli [40,41]. Given the increased IL-8 levels, these transcription pathways were evidently stimulated by both LPS and the protein extracts. Moreover, given that both LPS-treated and gliadin-treated intestinal cell lines were previously reported to induce COX-2 expression [21,41,42,43], it is feasible that COX-2 was similarly expressed in the present study. Interestingly, it was shown that COX-2 expression was induced in HT-29 cells (in response to the mycotoxin, deoxynivalenol) but that this did not result in an increased expression of PGE2, which remained unchanged [44]. The absence of increased PGE2 levels in the present study may, similarly, suggest an effect attributable to post-transcriptional mechanisms that are necessary for the expression of this compound.

In the present study redistribution of the TJ proteins, occludin, and more specifically the loss of ZO-1, were evident after a 24-h exposure to 40 µg/mL wheat proteins, corroborating previous observations [24,25,45]. Aside from gliadin-induced stimulation of MAPK- and NF-kB-dependent production of cytokines, which impact on TJ permeability, gliadin has also been shown to induce an increase in zonulin, in turn associated with actin polymerization, downregulation, and/or redistribution of ZO-1 and occludin and increased membrane permeability [24,25,26,46]. Interestingly, LPS (100 ng/mL) after 2 h was previously shown to dramatically reduce the stain for ZO-1 at the intercellular junctions in a disproportionate manner, with a negligible effect on occludin distribution. This indicated that ZO-1 was lost in a rapid manner under relatively low concentrations of LPS [45]. Instead, occludin distribution at the TJ was then also reduced disproportionately at 500 ng/mL LPS, coinciding with a dephosphorylation of the threonine residues and diffuse staining, indicating the deposition in the cytoplasm [45]. In the present study, staining of occludin proteins in the Caco-2 cells exposed to the landrace flour mixes was disproportionate. However, the staining was distinct, which contrasted with the diffuse and faint staining within the cytoplasm of the cells exposed to two of the modern flour mixes. The diffuse staining suggested an increased dephosphorylation and redistribution of occludin. The Caco-2 cells exposed to the highest gluten strength flour mix appeared visibly more damaged and leaching of occludin from the cells is suggested to have occurred. Previous research using Caco-2 cells exposed to 1 mg/mL gliadin for either 1 h [25] or 4 h [24] also showed a mosaic staining of ZO-1, in which there were areas with reduced or loss of ZO-1 staining and areas in which no significant changes were detected. With the exception of Caco-2 cells exposed to single modern wheat flour mix, ZO-1 staining was not evident, suggesting that all wheat proteins induced the early loss of ZO-1. While ZO-1 displacement, per se, is not sufficient to cause a barrier defect, in combination with occludin displacement and actin polymerization, TJ disassembly is suggested to be likely [46]. Although permeability of Caco-2 was not measured in the present study, the microscopic images visually testify not only to increased permeability but cell breakage, particularly evident after exposure to modern genotype flour mixes. Given that TJ barrier permeability is shown to be increased, not only in CD patients but also in individuals suffering with WA and NCWS [47], further study of the causative factors is important.

In the present study, migration of U937 monocytes toward the Coco-2 monolayer in the 2D co-culture was corroborated by U937 migration in the more physiologically relevant 3D co-cultures [27]. CD14 staining of the glycolipid-anchored membrane glycoprotein expressed on U937 cells enabled the identification of the pro-inflammatory-induced activation of the latter, which in the present study were greater following exposure to the higher gluten strength modern flour proteins. Interestingly, incubation of CD14 activated monocytes isolated from CD patients with cultured epithelial cells, resulting in an increase in TJ permeability, lower levels of occludin, and a mosaic expression pattern of ZO-1, an effect that was not evident following incubation with monocytes of healthy donors [48]. Given the potency of activated monocytes, the potential contribution of activated U937-monocyte-induced effects on Caco-2 cell TJ proteins cannot be excluded. Of interest, increases in soluble or serum CD14, evident following the shedding of CD14 from the surface of activated monocytes, is described as a nonspecific marker of monocyte activation in innate immunity [49]. Of relevance to the present study, NCWS patients were reported to have increased serum CD14, suggesting a link between the intestinal epithelial cell damage and the acute systemic immune activation [4].

Differentiation of activated CD14 U937 monocytes into CD11b identifiable macrophages was similarly more evident for the modern variety flour mixes. Wheat protein was shown to be pro-inflammatory, similar to phorbal ester, which induced U937-macrophage differentiation [28,29]. Regarding wheat proteins, the effects of gliadin were previously reported to extend to macrophages, central to the innate immune response and present in the lamina propria. Gliadin activation of macrophages in vitro was found to upregulate expression of a panel of inflammatory genes and result in the secretion of inflammatory cytokines [50].

Though the intestinal models do not precisely reflect the in vivo situation, the present results collectively show an increased cytotoxicity and immunogenic potential of the modern genotype flour mix proteins in comparison to the old genotypes on monocultures and 2D and 3D co-cultures, respectively. The present study was preliminary but highlighted that the development of common wheat genotypes with increased GS for improved baking potential, with requested specifications by processing industries, coincided with greater potential harm to the intestinal mucosa. Though total proteins were shown to impact on the cells, in vivo the proteins are subjected to partial digestion prior to arrival at the small intestine, and future research will necessitate investigating whether more easily digestible genotypes (with varying GS) impact differently on cell lines.

## 4. Materials and Methods

### 4.1. Wheat Samples

Old *Triticum aestivum* heritage wheat flour mixes, produced for commercial use, were obtained from two locations in the Emilia-Romagna region (Italy). The two source farms were both certified for organic farming and incorporated biodynamic principles. The flour mixes in both farms were produced from the same five heritage genotypes that were cultivated in mixtures during the 2017–2018 season. The genotypes were Andriolo, Gentil Rosso, Verna, Frassineto, and Inalletabile with release dates of approximately 1945, 1900, 1953, 1932, and 1920, respectively (National Italian Varietal Register of the Agricultural Ministry). Artisan-based, whole-grain, stone-ground flour was obtained. The two heritage flour mixes are from here on referred to as “Lacollina_A” and “Virgo_A”.

The modern flour mixes were obtained from genotype mixes, cultivated under conventional agricultural farming practices in the 2017–2018 season. The modern flour mixes (comprised of undisclosed genotypes) were obtained from the mill “Mulino di Granaceto”, forming part of the “Progeo Molini” co-operative in Emilia-Romagna. The flour mixes were specifically selected based on end-use quality classification representing confectionary wheat (“frumento biscottiero”), ordinary bread-making wheat (“frumento panificabile”), and improved wheat (“frumento di forza”). The wheat flours, based on the different rheological properties, are from here on referred to as “Biscottiero_M”, “Panificabile_M”, and “Diforza_M”, respectively.

### 4.2. Quality Parameters

Using the flour Infratec™ 1241 Grain Analyser (FOSS, Analytical A/S, Hillerød, Denmark), near-infrared spectroscopy, moisture content, ash crude protein, and wet gluten were measured. Alveograph (deformation energy, W [10^−4^ J], maximum pressure, P (mm), and extensibility, L (mm)) parameters (Alveograph MA 82, Chopin Technologies, Villeneuve-la-Garenne, France) were measured according to the standard AACCI (American Association of Cereal Chemists) Approved Method 54-30.02 [51]. Alveograph doughs were prepared at constant water absorption according to the manufacturer’s guide.

### 4.3. Protein Fraction Extraction and Quantification

Protein fractions were extracted from 100-mg flour samples by the Osborne procedure, according to the method originally described by Lookheart and Bean [52] on the basis of solubility, resulting in the sequential extraction of albumins (distilled water), globulins (0.5 M NaCl), gliadins (70% ethanol), and glutenins (50% iso-propanol and 1% dithiothreitol). The albumin and globulin fractions were combined, as were the gliadin and glutenin fractions, respectively. An aliquot of 100 µL of both albumin/globulin and gliadin/glutenin fractions were added to 80% acetone overnight at −20 °C and the precipitated proteins suspended in filtered (0.45 µM) 8 M urea. The albumin/globulin and gliadin/glutenin fractions were also solubilized independently and quantified for experimental procedures requiring separate use of the two fractions.

The quantification of proteins extracted from the five different flour varieties was carried out by spectrophotometry (at 450 nm) using the Pierce Rapid Gold BCA Protein Assay Kit (Thermo Fisher Scientific^®^,Waltham, MA, USA), with bovine serum albumin as reference standard, according to the manufacturer’s instructions.

Protein samples (20 µg) of the albumin/globulin and gliadin/glutenin fractions, respectively, were separated by sodium dodecyl sulfate (SDS)-polyacrylamide gel electrophoresis (PAGE). Pre-prepared 2× Tris-glycine SDS sample loading buffer (Novex^®^, Thermo Fisher Scientific^®^,Waltham, MA, USA) and 5% mercaptoethanol were added to each sample. The samples were denatured at 98 °C for 5 min, loaded onto the Tris-glycine, graduated 4–12% (Invitrogen^®^, Carlsbad, CA, USA) polyacrylamide gel, and allowed to run at 120 V until completion. Gels were stained with a solution of coomassie blue (Sigma-Aldrich^®^, St. Louis, MO, USA) 0.1% coomassie blue in 10% acetic acid) for 1 h under agitation. Destaining was performed in a solution of methanol:acetic acid:water, ratio of 3:1:6, overnight. Thereafter, the gel was washed in 2% glycerol solution to remove the excess acetic acid before image analysis and subsequent drying. The imageJ program Fiji (Wayne Rasband, National Institute of Mental Health, Bethesda, MD, USA) was then used to analyze the stained gels and compute the relative distances between the various bands of each sample to assign the correct kDa values.

### 4.4. Cell Model Systems and Growth Maintenance Conditions

The Caco-2 human epithelial cell line (ATCC®, American Type Culture Collection, HTB-37^TM^), obtained from colorectal adenocarcinoma, the U937 pro-monocytic, human lung myeloid leukemia cell line (ATCC® CRL-1593.2) and L929 mouse fibroblasts (ATCC ®-CCL1) were cultured as described previously in Truzzi et al. [53]. Stock cultures of all cell lines were maintained at 37 °C in a humidified atmosphere containing 5% CO_2_ in tissue culture flasks (75 cm^2^; BD Biosciences, Franklin Lakes, N J, USA), and the culture medium changed every two days. Prior to experimentation, the Caco-2 cells were trypsinized and cell density evaluated microscopically using a Bürker counting chamber.

### 4.5. Monoculture Experiments Using Caco-2 and U937 Cell Lines

Caco-2 cells were plated into 96-well tissue culture plates (10^5^ cells/well) in complete medium. After 24 h, cells were treated with total wheat protein. From the total concentrated total protein suspended in 8 M urea (Section 4.3), the correct aliquot of protein (to attain a concentration of 40 µg/mL) for the five flour samples, respectively, was added to Dulbecco’s Modified Eagle Medium (DMEM)). For the untreated controls (without protein extract), the cells were incubated in an equivalent concentration of urea (as the protein extracts) added to DMEM. The same procedure was used for the 2D and 3D co-culture experiments for all controls not containing wheat protein. Following the 24-h treatments, the medium was carefully aspirated and cell proliferation and vitality were measured. Additional experiments using the albumin/globulin fraction (40 µg/mL in DMEM), as well as the gliadin/glutenin fraction (40 µg/mL in DMEM), respectively, were similarly performed.

U937 cells were sown in a 12-well culture plate (15 × 104 cells/well). After 24 h, total protein (40 µg/mL in DMEM) was added to each of the five samples. The untreated control contained an equal volume of DMEM without protein extracts. Following the 24-h exposure, the medium was carefully removed by centrifugation at 1200 rpm for 5 min and tested for vitality using the trypan blue exclusion assay [53]. All monoculture experiments on both the Caco-2 and U937 cell lines were performed in triplicate.

### 4.6. Two-Dimensional Co-Culture Experiments Using Caco-2 and U937 Cell Lines

Boyden’s chambers, containing a cylindrical cell culture insert nested inside each well of the culture plate, were used for the Caco-2 and U937 co-culture experiments (Transwell, Costar, Cambridge, MA, USA). Caco-2 cells (10 × 10^4^) were plated at the base of each well and were adherent to the base of the culture plate. After a period of 24 h, the Caco-2 cells were treated with either LPS (1 µg/mL in DMEM) or the protein extracts from the five samples, respectively (40 µg/mL in DMEM). The untreated control contained the same volume of DMEM without protein. After the 24-h exposure period to the respective treatments, the inserts, each with a 0.4-µm filter at the base, were inserted. U937 monocytes (50 × 10^4^) in 200 µL complete Roswell Park Memorial Institute (RPMI) medium were added above the filter. After 4 h, the filter supports were removed and cell migration and cell vitality were determined in the U937 cells and Caco-2 cells, respectively. The trypan blue exclusion assay was used to determine the vitality of the Caco-2 cells [53]. All co-culture experiments were performed in triplicate.

### 4.7. Three-Dimensional Reconstituted Intestinal Equivalents Using Caco-2, U937, and L929 Cells

The 3D cultures were performed as described previously [53]. Cell-free 0.5 mg/mL collagen solution (1.35 mg/mL rat tail collagen type I in DMEM with 10% fetal bovine serum, FBS, and 1% Pen/Strep) was added to tissue culture inserts (Transwell, Costar, Cambridge, MA, USA) in 12-well plates. This pre-coated layer was overlaid with 1 mL of L929 fibroblasts (1 × 105/mL) together with U937 monocytes (3 × 10^4^/mL) mixed with collagen type I. After incubation (2 h) at 37 °C, Caco-2 cells (2 × 10^5^) were seeded onto dermal reconstructs and incubated at 37 °C with Caco-2 medium, added both on the upper and the lower part of the filter support. After five days, the cell models were treated with either 40 µg/mL of total protein from the five samples, respectively, or DMEM for the control. Exposure to the protein extracts was for 24 h. The cells were then fixed with formalin for 2 h at room temperature, dehydrated, and embedded in paraffin.

### 4.8. Cell Proliferation Measurements

Proliferative Caco-2 cells in monoculture were detected using the 3-(4,5-dimetiltiazol-2-il)-2,5-difeniltetrazolio (MTT) assay, according to the ISO 10993-5 International Standard procedure (ISO 10993-5, 2009). The method is based on the reduction of MTT by mitochondrial dehydrogenase of intact cells to produce purple formazan, determined by measuring the absorbance at 540 nm using a multiwell scanning spectrophotometer (Labsystems Multiskan MS Plate Reader, ThermoFisher Scientific), as described by Truzzi et al. [53].

### 4.9. Determination of Cell Vitality

Cell vitality of the U937 monocytes in monoculture, as well as the Caco-2 cells in 2D co-culture, was measured using trypan blue as reported in Truzzi et al. in 2020 [53]. In brief, cells were carefully resuspended in a 0.4% trypan blue (Gibco) solution. Vital cells were counted using the Countess^®^II FL (ThermoFisher Scientific, Waltham, MA, USA) and results expressed as a viability percentage of the control. Vitality of Caco-2 cells (10 × 105 cells/well) in co-culture were measured using phase contrast microscopy using an inverted microscope (Eclipse Ts2, Nikon) following exposure to LPS and protein treatment. The cells, resuspended in a 0.4% trypan blue (Gibco) solution, were visualized in the wells at a magnification of 40×.

### 4.10. Cell Migration Measurements

After the 4-h exposure to chemo-attractants emitted from Caco-2-treated cells in 2D co-culture, the filters containing migratory U927 cells were stained with MGG QUICK STAIN (Bio-Optica^®^, Mi, Italy) containing thiazine for 10 s. Thiazine-stained U937 cells from the filters were placed onto slides, to which 50 µL glycerin was added, and cells were counted using an optical microscope (Eclipse Ts2, Nikon,Tokyo, Japan). Cells in 10 optical fields were photographed for each filter. The average number of cells was estimated from the 10 optical fields.

### 4.11. Measurement of Interleukin-8 (IL-8) and Prostaglandin E2 (PGE2)

Following the 2D co-culture treatments as described above, the cell medium was analyzed for the level of IL-8 in the culture and supernatants were determined using IL-8 human ELISA kit (ThermoFisher Scientific) according to the instructions by the manufacturer. PGE2 was similarly determined in the cell medium using PGE2 monoclonal enzyme immunoassay (Cayman Chemicals, Ann Arbor, MI, USA) according to the manufacturer’s instructions.

### 4.12. Western Blot Analysis of Occludin Expression Levels

After the 24-h treatments of Caco-2 cells in 2D co-culture, the cell medium was washed with phosphate-buffered saline, PBS, and adherent cells were lysed on ice in radioimmunoprecipitation assay buffer (RIPA buffer) pH 7.5. Total protein was quantified according to the Pierce™ BCA Protein Assay Kit (Thermo Fisher Scientific). Total protein (40 μg) was analyzed under reducing conditions on Bolt™ 8%, Bis-Tris Protein Gel (Thermo Fisher Scientific^®^,Waltham, MA, USA) and blotted onto nitrocellulose membranes. The blots were blocked for 1 h with SuperBlock™ T20 (PBS) Blocking Buffer (Thermo Fisher Scientific^®^,Waltham, MA, USA) and incubated with anti-human Occludin (1:1000, Novus Biologicals, Centennial, CO, USA) and β-actin (1:1000, Invitrogen, Carlsbad, CA, USA) overnight at 4 °C. The membranes were then washed in PBS/Tween 20 and incubated with peroxidase-conjugated goat anti-mouse antibodies (1:505, Invitrogen) for 45 min at room temperature. Finally, membranes were washed and developed using the Pierce™ Fast Western Blot Kit, ECL Substrate (Thermo Fisher Scientific^®^,Waltham, MA, USA). The band intensity was quantitatively determined using ImageJ software Fiji (Wayne Rasband, National Institute of Mental Health, Bethesda, MD, USA) and occludin protein levels normalized to β-actin expression.

### 4.13. Immunohistochemical Analysis

Paraffin-embedded reconstituted 3D intestinal equivalents were rehydrated. Sections (4 μm thick) were stained with hematoxylin and eosin (H & E), as well as with thiazine (Bio-Optica^®^, Milan, Italy). Sections were stained with the following antibodies: Occludin (Novus), Zonulin (ZO-1, Thermo Fisher Scientific), CD14, and CD11b (GeneTex Inc., Irvine, CA, USA). Immunohistochemistry was performed using fast red chromogen according to the UltraTek Alk-Phos Anti-Polyvalent (permanent red) Stain Kit (ScyTek Laboratories, Inc., Logan, UT, USA). Negative controls were obtained by omitting the primary antibody.

### 4.14. Statistical Analysis

Statistical analysis was conducted using CoStat version 6.450 (2017) software (http://www.cohort.com). Significance was determined by one-way variance (ANOVA) and the Tukey–Kramer test was performed to determine any significant differences between treatments at *p* ≤ 0.05.

## Figures and Tables

**Table 1:** Technical and alveograph gluten parameters in three commercially available modern wheat mixes (denoted by M) and two landrace wheat mixes (denoted by A).

	Biscottiero_M	Panificabile_M	Diforza_M	Lacollina_A	Virgo_A
Ash (%)	0.59 b	0.60 b	0.61 b	1.06 a	1.25 a
Protein (%)	11.0 c	11.0 c	12.4 c	15.6 a	13.2 b
Moisture (%)	11.6 a	11.8 a	11.8 a	11.6 a	12.0 a
Wet Gluten (%)	19.7 b	20.3 b	22.5 b	26.4 a	24.4 a
Gluten strength W (10^−4^ J)	119 c	238 b	389 a	64 d	53 d
Extensibility P/L (mm/mm)	0.29 c	0.48 b	0.65 a	0.46 b	0.43 b

The designation of the letters a, b, c, and d represents significant differences between varieties as determined by one-way ANOVA at the 95% confidence level (*p* ≤ 0.05). P is the dough tenacity and L is the dough extensibility.

## Data Availability

The data that supported the findings of the present study are available from the corresponding author upon request.

## Supplementary Materials

The following are available online at https://www.mdpi.com/1422-0067/22/1/172/s1.

## Abbreviations

GS	Gluten strength
2D	Two dimensional
3D	Three dimensional
CD14	Cluster of differentiation 14
CD11b	Cluster of differentiation 11b
HMW-GluS	High molecular weight glutenin subunits
LMW-GluS	Low molecular weight glutenin subunits
CD	Celiac disease
WA	wheat allergy
NCGS	Non-celiac gluten sensitivity
NCWS	Non-celiac wheat sensitivity
ATIs	Amylase-trypsin inhibitors
P/L	gluten strength/extensibility
A + G	Albumins + globulins
LPS	Lipopolysaccharide
IL-8	Interleukin-8
PGE2	Prostaglandin E2
COX-2	Cyclooxygenase-2
TJ	Tight junction
ECM	Extracellular matrix
H & E	Hematoxylin and eosin
ZO-1	Zonula occludin-1
MAPK	Mitogen-activated protein kinase
NF-kB	Nuclear factor kappa-light-chain-enhancer of activated B cells
