# Supplementary material for: Pro-Inflammatory Effect of Gliadins and Glutenins Extracted from Different Wheat Cultivars on an In Vitro 3D Intestinal Epithelium Model
Source: Int J Mol Sci. 2020 Dec 26;22(1):172. doi: 10.3390/ijms22010172 (PMC7795490; doi:10.3390/ijms22010172)
Supplement: Supplementary file 1 [file ijms-22-00172-s001.pdf]

Supplementary Material

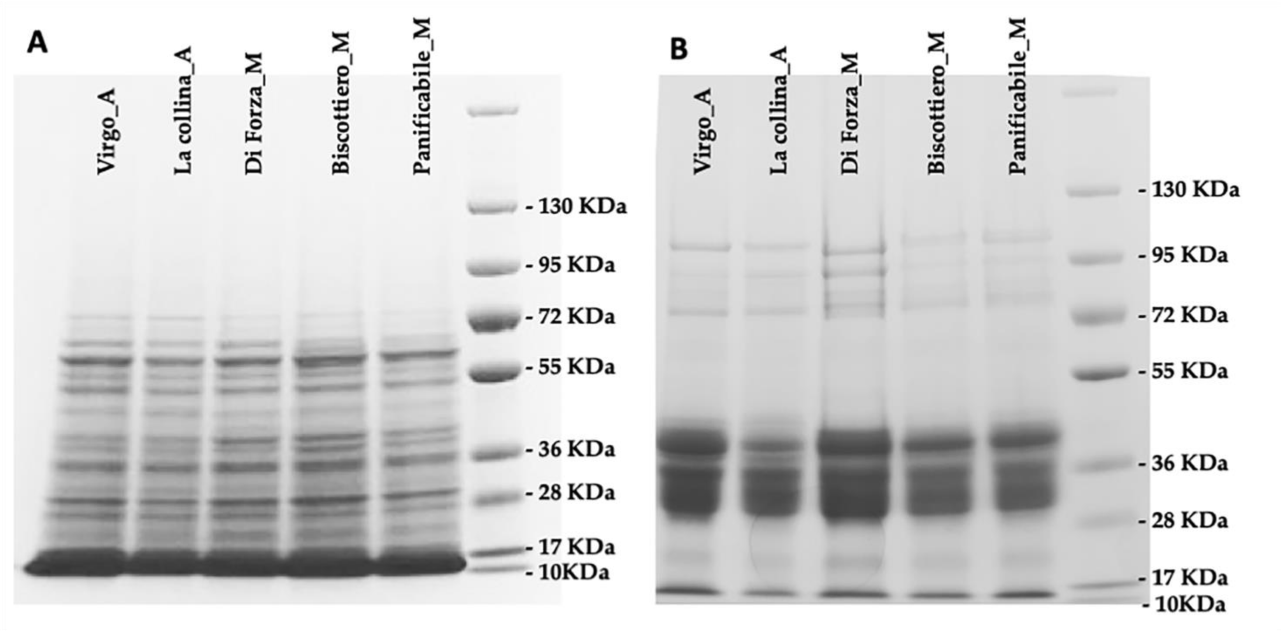

Figure S1. Sodium dodecyl sulfate (SDS) - polyacrylamide gel electrophoresis (PAGE) of (A) the albumin/globulin fraction and (B) the gliadin/glutenin fraction (20 μg protein per lane) from three commercially available modern wheat mixes (denoted by M) and two heritage wheat mixes (denoted by A).
